# Extrafoveal attentional capture by object semantics

**DOI:** 10.1371/journal.pone.0217051

**Published:** 2019-05-23

**Authors:** Antje Nuthmann, Floor de Groot, Falk Huettig, Christian N. L. Olivers

**Affiliations:** 1 Psychology Department, School of Philosophy, Psychology and Language Sciences, University of Edinburgh, Edinburgh, United Kingdom; 2 Institute of Psychology, University of Kiel, Kiel, Germany; 3 Department of Experimental and Applied Psychology & Institute for Brain and Behaviour, Vrije Universiteit, Amsterdam, The Netherlands; 4 Max Planck Institute for Psycholinguistics, Nijmegen, The Netherlands; 5 Centre for Language Studies, Radboud University, Nijmegen, The Netherlands; SWPS University of Social Sciences and Humanities, POLAND

## Abstract

There is ongoing debate on whether object meaning can be processed outside foveal vision, making semantics available for attentional guidance. Much of the debate has centred on whether objects that do not fit within an overall scene draw attention, in complex displays that are often difficult to control. Here, we revisited the question by reanalysing data from three experiments that used displays consisting of standalone objects from a carefully controlled stimulus set. Observers searched for a target object, as per auditory instruction. On the critical trials, the displays contained no target but objects that were semantically related to the target, visually related, or unrelated. Analyses using (generalized) linear mixed-effects models showed that, although visually related objects attracted most attention, semantically related objects were also fixated earlier in time than unrelated objects. Moreover, semantic matches affected the very first saccade in the display. The amplitudes of saccades that first entered semantically related objects were larger than 5° on average, confirming that object semantics is available outside foveal vision. Finally, there was no semantic capture of attention for the same objects when observers did not actively look for the target, confirming that it was not stimulus-driven. We discuss the implications for existing models of visual cognition.

## Introduction

Visual acuity declines systematically from the central fovea (~1° to either side of fixation) through parafoveal vision (until ~1–5° eccentricity) into the periphery (>5°) and imposes important constraints on scene perception and visual search [1]. Despite its lower spatial resolution, extrafoveal vision is crucial to guiding attention to objects that are to be fixated next [2–4]. An unresolved question is what information is available for guiding the next eye movement–that is, whether extrafoveal information processing is restricted to low-level, visual features, or whether it extends to high-level, semantic properties. It has been shown that extrafoveal objects that are visually salient are preferentially selected for attention and fixation[5, 6], but whether objects in extrafoveal vision can also be selected on the basis of object meaning has caused a good deal of controversy in the literature (see refs. [7] and [8] for reviews). Here, we provide additional evidence that extrafoveal semantic object information can capture attention and drive the next eye movement.

A common way to study the availability of object meaning beyond the fovea has been to manipulate the semantic relationship between an object and the scene background in which it is located, such that it is either congruent or incongruent with that scene. The underlying reasoning is that an incongruent object (e.g., an octopus in a farm scene) is more informative than a congruent one (e.g., a tractor in a farm scene), and so should attract attention [9, 10]. If extrafoveal processing of semantic congruencies takes place, then incongruent objects should be fixated earlier in time than congruent ones, as assessed by the time it takes to select the critical object for fixation (i.e., the latency to first fixation on the object), and search times should be shortened accordingly. However, results have been mixed, with evidence for [11–16] and against [17–21] prioritized processing. Furthermore, only one of these studies has reported evidence for *immediate* extrafoveal attentional capture by object-scene semantics–that is, upon first fixation [15]. Here, the average amplitude of the saccade into the critical object was more than 7°, suggesting that viewers could process semantic congruencies based on peripheral information obtained in a single fixation.

Although there is thus some evidence that semantically inconsistent information can be detected in extrafoveal vision, several authors have argued that the incongruency effect may only emerge for scene material in which the incongruent objects are also *visually* more salient than the congruent ones (see refs. [22] and [8] for reviews). Subsequent studies that explicitly controlled or manipulated visual salience have shown mixed results as well [12, 16, 18, 20, 21, 23]. Therefore, it remains an open question to what extent visual salience can explain the positive findings in the literature. Besides saliency, other factors may also contribute to the appearance of the incongruency effect, most notably image clutter and contextual guidance. It has been suggested that scenes that are visually more complex and cluttered produce more lateral masking (crowding) [24], which may in fact hinder semantic analysis in extrafoveal vision [12, 22]. In this respect it is noteworthy that the original Loftus and Mackworth study [15] used relatively sparse line drawings of scenes. Moreover, when searching the scene for a particular target object, observers use their knowledge about the likely positions of consistent targets to drive their eye movements [18]. Depending on the scene material, this may negate or even reverse any attentional guidance towards inconsistent target objects.

A solution for the problems mentioned in the previous paragraph is to use sparse displays that consist of individual objects against a homogeneous background. Semantic relatedness is then defined by the relationship between one of the non-target objects in the display and a sought-for target object, rather than the association between the object and the rest of the scene. Working with arrays of standalone objects rather than scenes avoids contextual guidance and allows one to reduce, or to control the amount of visual clutter in the stimulus material. At the same time, visual salience relative to the background is less of an issue because the objects that are manipulated on the semantic dimension are not embedded in scenes. However, it is important to ensure that any effects of semantics are not driven by visual appearances. Specifically, confounding semantic relatedness with visual similarity must be avoided, and objects of a certain type need to be matched on low-level visual parameters like luminance and visual complexity.

Two earlier studies have used displays with standalone objects to investigate semantic influences on the selection of stimuli for attention in visual search [25, 26]. These studies were not designed to investigate extrafoveal attentional capture by object semantics, but they report results that speak to the issue. Using photographs of everyday objects, Moores, Laiti, and Chelazzi [25] found that the first saccade went significantly more often to objects that were semantically related to the target than to unrelated objects, despite these objects being presented approximately 6° from fixation. Items were chosen by the experimenters for their low similarity, plus each item served as its own control in displays where it was not associated with the target. However, with only twelve pairs of semantically related objects, this design implies that objects were repeated several times throughout the experiment, possibly leading to learning of the relationships, or familiarity effects. A study by Belke, Humphreys, Watson, Meyer, and Telling [26] also found effects of object category on the first eye movement. At approximately 7.5° eccentricity, the objects in this study were also clearly presented extrafoveally. Belke et al. furthermore performed a rating study to control for visual similarity. However, stimuli consisted of line drawings, which are reduced depictions of real-world objects. Moreover, here too items were repeated multiple times, making the semantic relationship rather predictable.

Recently, we investigated the effects of semantic information on visual selection using displays that circumvent many of the problems discussed above [27, 28]. In these experiments, observers were asked to look for a verbally described and auditorily presented target object. The visual displays contained a number of standalone objects, and relationships were defined by the sought-for target object rather than a scene. The target-absent displays were the crucial ones as they contained an object that was semantically related to the word, an object that was visually related to the word, plus one or two unrelated objects. Rather than line drawings, photographs of real-life objects were being used and initially placed in peripheral vision. The visual stimuli were highly controlled on both visual and linguistic features. Additionally, semantic and visual relatedness were dissociated [29]. Finally, every display was unique, to avoid repetition effects.

The goal of these studies was to uncover how semantic and visual influences on attention differentially developed relative to each other over time throughout the search process. For this purpose, the stimulus timing was varied by presenting the search display either *prior* to the spoken target word (preview, following the visual world literature [30]) or *following* the target instruction (no preview, as in the standard visual search literature). To assess the time course of any biases towards visually or semantically related objects, the main analysis divided up the trial into twenty 100 ms time bins. For each time bin, the proportion of time that people spent fixating a particular object in a bin was computed. It was consistently found that significant biases developed in proportion fixation time toward both visual and semantic competitors. The visual bias was stronger than the semantic bias and started earlier. Additional analyses showed that the number of fixations on associated competitors was increased, both the number of fixations that came in from elsewhere and the number of fixations remaining within the object.

These aggregate measures of proportion fixation time and number of fixations do not allow for firm conclusions about the role of extrafoveal vision in object prioritization. To fill this gap, in the present article we report a set of measures that were not in the orginal publications, and that were designed to index extrafoveal processing of objects’ perceptual and semantic information, plus potential differences in foveal processing. First, the *probability of immediate object fixation* will tell us whether semantic information can exert an immediate effect on eye-movement control [15]. Second, we directly assess the *latency to first fixation* on objects of a given type [20, 23] as a measure of whether semantic information has a temporal advantage in extrafoveal vision. Third, as an additional index of extrafoveal processing we report the *incoming saccade amplitude* [18], representing the empirical retinal eccentricity between successive fixations. Fourth, we report *first-pass gaze duration* as a standard measure of foveal object processing [18, 22]. We limited our analyses to the no preview conditions, since any preview would allow observers to fixate objects before hearing the target description. In addition, we included a condition of the experiment reported in [28] in the analyses, in which the word that the observers heard was not relevant to the search task. From a methodological point of view, this accessory condition provides an additional control for the possibly confounding effects of low-level visual properties. Here, the presented visual objects were not relevant to the search task and thus, any prioritization of semantically related objects would be indicative of low-level bottom-up factors. Finally, we chose to apply linear mixed-effects models (LMM) [31] and generalized linear mixed-effects models (GLMM) [32], which have many advantages over analyses of variance (ANOVA) [33]. While there is a strong movement towards replacing ANOVA with (G)LMM in psycholinguistics [31, 32], researchers in scene perception and visual search have only just begun to exploit mixed models [34, 35]. Using mixed models allows us to capture variance attributed to the randomness of participant and item sampling simultaneously within a single analysis [36]. Besides, we avoid information loss due to prior averaging over subjects or items. In addition, mixed models can handle incomplete and unbalanced data, an inherent feature of many eye-tracking studies. A further specific advantage in the present context is that the fixed-effect coefficients are directly interpretable as they describe differences between visual and semantic biases. This new type of analysis together with previously unreported measures provides solid evidence for the claim that object semantics can indeed be processed rapidly outside the fovea and drive the first eye movement.

## Method

The data are from three earlier experiments [27, 28], which we analysed on different aspects. In the present report, Experiment 1 and 2 correspond to Experiments 1 and 2 from ref. [27]; Experiment 3 is the experiment from ref. [28].

### Participants

A total of 68 Dutch native speakers participated for course credits or monetary compensation. Twenty participants (aged 18–26, average 20.6 years, 2 males) took part in Experiment 1. Experiment 2 and Experiment 3 each had 24 participants (Experiment 2: aged 18–29, average 21.3 years, 8 males; Experiment 3: aged 17–37, average 21.6 years, 4 males). In Experiment 1, two participants were replaced because of calibration failure or technical problems during eye movement recording. In Experiment 2, three participants were replaced: two as they scored overall less than 90% correct and one because of technical failures in data acquisition. All participants gave informed consent, reported no history of colour blindness and/or language disorders, and participated only once. Written informed consent was obtained from all participants, and all studies were conducted in accordance with the Declaration of Helsinki and approved by the Scientific and Ethical Review Board of the Faculty of Behavioural and Movement Sciences at the Vrije Universiteit Amsterdam (approval letter of 12-10-2010, renewed 02-01-2017, file number VCWE-2016-215).

### Apparatus

Stimuli were presented in OpenSesame [37] (version 2.7.3 in Experiment 1 and 2 and version 2.9.7 in Experiment 3). Words were presented through headphones (Sennheiser HD202) connected via a USB Speedlink soundcard. In Experiments 1 and 2, participants were tested on a Pentium IV computer (2.3GHz) with a 21-in. SVGA monitor (refresh rate of 100 Hz, resolution of 1024 × 768 pixels). The distance from the chinrest to the screen was 56 cm. An Eyelink 1000 Tower Mount system (SR Research Ltd., Canada) with a temporal and spatial resolution of 1000 Hz and 0.01° respectively was used to track the right eye. In Experiment 3, participants were tested on a HP ProDesk 600 G1 CMT computer with a Samsung Syncmaster 2233RZ monitor (refresh rate of 120Hz, resolution of 1680 × 1050 pixels) with a distance of 70 cm between the monitor and chin rest. An Eyelink 1000 Desktop Mount with a temporal and spatial resolution of 1000 Hz and 0.01° respectively was used to track the left eye.

### Materials

**Fig 1:**
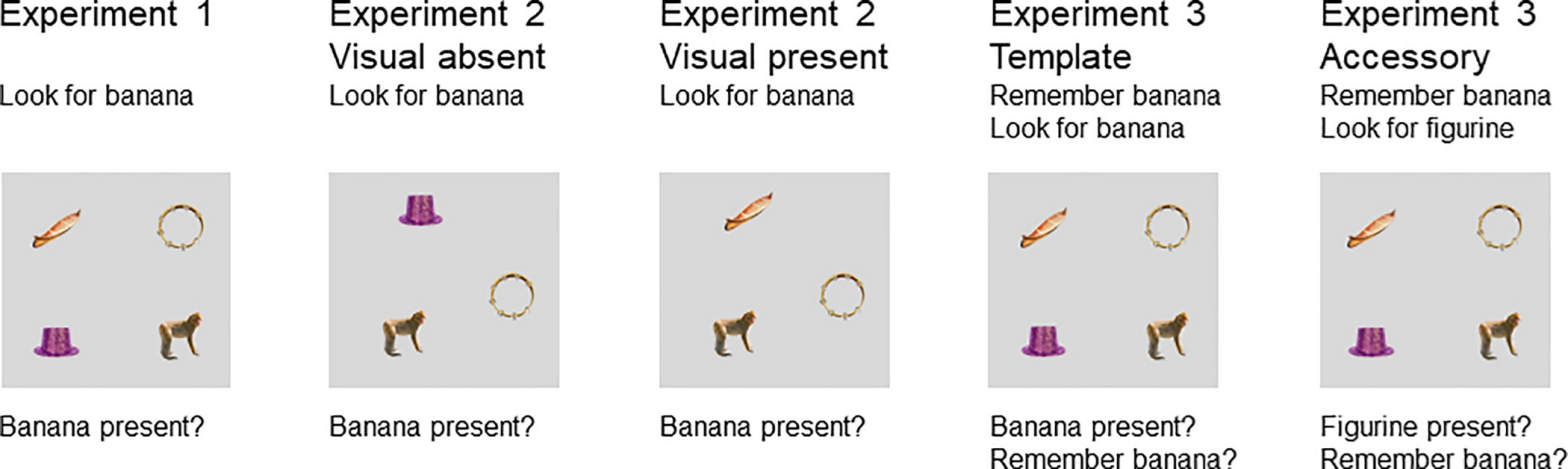
An example search display in the different experimental conditions across three experiments. On this particular trial, the spoken target word was “banana.” The relevant displays contained no target but included objects that were semantically related to the target (“monkey”) or unrelated (“hat” and “tambourine”). In most experiments (except for Experiment 2 in the visual absent condition) these trials also contained a visually related object (“canoe”). In all but one condition (i.e., the accessory condition in Experiment 3), participants indicated whether the target object (“banana”) was present or absent in the display. Pictures are from the license-free Hemera Photo-Object database (Vols. I, II, & III; Hemera Technologies Inc.).

In all experiments, there were 120 target-present and 120 target-absent trials. On the target-present trials, the spoken target word referred to an object in the display, while there was no deliberate relationship to other objects. The target-absent trials were the critical trials as they contained objects that were either semantically or visually related to the word. Importantly, semantically related objects were not visually related (and vice versa). For example, if the target word was “ruler”, the semantically related object was a picture of a hole puncher, and a picture of a comb established visual similarity; see Fig 1 for another example display. Pictures were mostly taken from the Hemera Photo-Object database (Volumes I, II, and III). Specifically, we started with a subset of semantically related object pairs from the POPORO stimulus set [38]. We then extended this set with both semantic and visual relationships, taking inspiration from earlier work [39, 40]. Similarity ratings were obtained from 61 naïve participants who were native speakers of Dutch (semantic similarity ratings: 30 participants, visual similarity ratings: 31 participants). In the rating studies, participants indicated on a 11-point scale how much the depicted object and the object that the spoken word was referring to “had something to do with each other, i.e., shared something in meaning or function” (semantic rating study) or how much they “looked alike” (visual rating study). Moreover, different object categories were controlled for several visual and psycholinguistic factors such as luminance, visual complexity, object size, word frequency, and naming agreement. For example, for each object two measures of object size were calculated: the radius of the smallest fitting circle around the object (as a measure of the object’s spatial spread) and the total number of pixels making up the object (as a measure of overall surface size). In the Dutch rating studies, we evaluated 520 pictures paired with 130 words. Based on the various ratings, the 100 best configurations were selected for the final stimulus set [29], which was extended by another 20 trials for the present experiments [27]. A complete list of stimuli and the details on all measures are provided in refs. [27] and [29]. The stimulus set, along with the norms and ratings, is available via the Open Science framework (https://osf.io/6vdys/?view_only=541cd6d599a74f4a99c7411e8cd60b4a).

In Experiments 1 and 3, the target-absent trials always included a semantically and a visually related object, along with two unrelated objects. In Experiment 2, only three pictures of objects were shown. The semantically related object was either presented together with two unrelated objects (visual absent condition) or together with a visually related and an unrelated object (visual present condition). Fig 1 shows an overview of the design for all three experiments.

In Experiments 1 and 3, the pictures were presented at four fixed positions on the screen, one in each quadrant of the display. The horizontal, vertical and diagonal distances from the centre of the picture to the fixation cross were 8°, 6° and 10° respectively. Thus, the objects’ centre-to-centre distance was 16° horizontally, 12° vertically and 20° diagonally. Regions of interest (ROI) were defined as squared areas of 8° and the radius of the smallest fitting circle around each object was on average 4° (SD = 0.36°). Thus, the smallest distance from the fixation cross to the edge of the ROI was 4° and to the edge of the smallest fitting circle on average 6°. In Experiment 2, the pictures were presented equidistant to each other. Their midpoints were situated on an imaginary circle around central fixation, with a radius of 7°. Thus, the centre-to-centre distance for the object pictures was 12°. The ROI were circular (radius of 4°, centred on the object). This was equal to the averaged radius of the smallest fitting circle around each object. This means that the closest edge of each ROI was 3° away from fixation cross. Thus, in all experiments the pictures were initially presented in extrafoveal vision. In all experiments and on all trials, it was randomly determined which picture occupied which position.

Words were recorded by author FdG. The spoken words could be one to four syllables long (target-absent trials: average duration 587 ms and range 327–926 ms; target-present trials: average duration 589 ms and range 274–950 ms). Trials were never repeated during the experiment, so each object and word was only presented once. All displays had the same grey background (RGB values: 230, 230, 230). The specific stimulus items were randomized and counterbalanced per two (Experiments 1 and 3) or per eight (Experiment 2) participants.

### Design

In all experiments, only the target-absent trials were analysed as they contained the different word-picture relationships. The full design of Experiment 1 was a 2 × 2 within-participants design with Trial Type (target absent vs. present trials) and Condition (no preview vs. preview) as factors. Condition was fully counterbalanced both within and across subjects in an ABAB design. Trial Type was mixed within blocks (50% each). For the present purposes, only the condition in which the spoken word was presented prior to picture onset (no preview condition) was examined because it allowed us to measure attentional capture time-locked to visual display onset. Experiment 2 had a 2 × 2 × 2 within-participants design with Trial Type (target absent vs. present trials), Condition (no preview vs. preview) and Presence of Visually Related Picture (present vs. absent) as factors. Condition was blocked in a counterbalanced ABAB design, whereas Trial Type and Presence of Visually Related Picture were mixed within blocks (50% target present trials, 25% target absent with a visually related picture and 25% target absent trials without a visually related picture). Again, only the no preview condition was considered, both for the condition where the visually related picture was present and where it was absent. The “visual absent” condition was included to see whether the presence of visual competition in the display affects the semantic effects and therefore serves as a control.

Experiment 3 used a 2 × 2 within-participant design with Trial Type (target absent and target present) and Task Relevance (template and accessory) as factors. Trial Type was mixed within blocks (50%), whereas Task Relevance was blocked in a counterbalanced ABABAB design. In the template condition, people had to memorize and search for the word, which implies that the word was relevant for the search task. Therefore, the template condition was comparable to the no preview condition in the other experiments. In the accessory condition, people were asked to memorize the word but to search for another object (a plastic figurine). Thus, in this condition the word was irrelevant for the search task.

### Procedure

In Experiments 1 and 2 there were four blocks of 60 trials each. In both experiments, each trial started with a drift correction which was triggered by a manual response when the participant fixated on a cross in the middle of the screen. After the manual response, the screen turned blank for 600 ms. In the no preview condition, the only condition we consider here, this was followed by an auditory presented word describing the target. Then, 2,000 ms after word onset the search display was presented. Participants had to indicate as fast and accurately as possible whether the verbally described object was present or absent in the visual display by pressing “*X”* or “*M”* on the keyboard (counterbalanced across participants). After the button press participants heard a click. The pictures however remained on the screen for another 1,000 ms and were then replaced by a blank screen. During this 1,000 ms period, eye movement recording continued. A new trial started after 600 ms.

In Experiment 3 there were six blocks of 40 trials each. Each trial started with a drift correction that was triggered by the space bar. A blank screen was then presented for 600 ms, which after a spoken word was presented through headphones. In the template condition this word was relevant for the search and for the memory task, whereas in the accessory condition people were instructed to memorize the word and search for a plastic figurine. A search display followed 2,000 ms after word onset. Participants used the keyboard to indicate whether the target was present (“J”) or absent (“N”). After the response they heard a click, and the search display stayed on the screen for another 1,000 ms. Then, as a memory test, a spoken word was presented again. Participants had to indicate whether this word was similar (“S”) or different (“D”) than the word they had heard before the search display. After a blank screen for 600 ms, a new trial began.

In all experiments people received two practice trials. Feedback was given only during the practice trials.

### Data processing and (G)LMM analyses

For a given experiment, the SR Research Data Viewer software was used to convert the eye-tracking raw data into a fixation report and an object-based interest area report. Those data were processed further and analysed using the R system for statistical computing (version 3.2.3; R Development Core Team, 2015) under the GNU General Public License (Version 2, June 1991). Trials with incorrect responses were removed. Data were not averaged, and analysed at the level of individual observations instead.

(G)LMM were fit to the data using the (g)lmer program of the lme4 package [41] (version 1.1–12) supplied in R, with the bobyqa optimizer (lmer) or a combination of Nelder-Mead and bobyqa (glmer). LMMs were estimated using the restricted maximum likelihood (REML) criterion, which is the default model-fitting approach [42]. GLMMs were fit by Laplace approximation. For our binomial GLMMs, we chose a logit link function, which is the default for glmer. For the (G)LMMs we report regression coefficients (*b*) and their standard errors (*SE*) along with the corresponding *t*-values (LMM: *t* = *b*/*SE*) or *z*-values (GLMM: *z* = *b*/*SE*). For GLMMs, *p*-values based on asymptotic Wald tests are additionally provided. For LMMs, a two-tailed criterion (|*t*| > 1.96) was used to determine significance at the alpha level of .05 [31].

LMMs were used for analysing continuous response variables, specifically the latency to first fixation on the object, the incoming saccade amplitude, and gaze duration. In the corresponding data matrix, each trial and/or item display was represented with as many entries as there were objects in the display (i.e., three or four). Analysis of our continuous response variables requires that a given object received at least one fixation in the trial. For objects that were never fixated, these dependent variables were coded as missing values (NA for ‘not available’). Objects with missing values were omitted for analysis.

To evaluate the effect of Object Type for each continuous response variable, we used treatment contrasts in which the semantically related objects served as the reference group. Consequently, the intercept for the fixed effect “Object Type” estimates the mean value for semantically related objects. The two slopes estimate the difference between unrelated and semantically related objects (unrelated-sem) and between visually related and the semantically related objects (vis-sem). The first difference score (unrelated-sem) describes any disadvantage of unrelated objects over semantically related objects, which is equivalent to an advantage of semantically related objects over unrelated objects. For a given trial, the two unrelated objects were combined to one unrelated variable. The only exception was the ‘visual absent’ condition in Experiment 2, in which there was no visually related object such that the two unrelated objects were evaluated separately. The second difference score (vis-sem) describes any additional advantage of visually related over semantically related objects. The actual coefficient for one of the other conditions can be derived by summing the difference score coefficient for this condition and the intercept.

We also investigated whether the direction of the very first saccade in the display was guided by object information in extrafoveal vision. The first saccade did not always land on one of the objects (range: 24.5% - 39.7% across experimental conditions). Favouring conservative hypothesis testing, we did not assign the first saccade to the closest object. To test our hypotheses, we analysed the probability of immediate fixation, which is a categorical dependent variable. When adopting the above analysis scheme, the probability of immediate fixation may then be assessed through a binary variable; in a given trial, the object representing a given Object Type (unrelated, visually related, semantically related) was selected with the first saccade (1) or not (0). However, only one of the three or four objects in the display can be fixated first. Consequently, if the dependent variable is ‘1’ for one of the objects, it is necessarily ‘0’ for the remaining objects. Thus, the observations for different objects within the same trial are not independent, which can lead to an underestimation of standard errors, which in turn can inflate the Type I error rate [43].

Therefore, we chose to estimate a separate, intercept-only GLMM for each contrast of interest. At a theoretical level, our a priori hypothesis concerned the difference between semantically related objects and unrelated objects. To exploit the full design of the studies, we additionally compared semantically related and visually related objects. For each experiment and/or experimental condition, the first analysis was based on trials in which the very first saccade was directed to either the semantically related object or an unrelated object. The second analysis was based on trials in which the first saccade was directed to either the semantically related object or the visually related object. For a given analysis, a binary response variable (0; 1) was created to distinguish between the two object types. In our intercept-only GLMMs, the intercept estimates the proportion of cases for which the response variable is coded with “1” in logit space. For both analyses, semantically related objects were coded with “1”. This allowed for testing whether the probability of immediate fixation was higher for semantically related objects compared to unrelated objects (analysis 1) and whether it was lower for semantically related objects compared to visually related objects (analysis 2).

In binomial logit mixed models, the parameter estimates are obtained on the log-odds or logit scale, and thus represent the log odds of selecting a particular competing object [43]. A logit of 0 corresponds to a probability of 0.5. Thus, if the fixed-effect estimate (*b*) for the intercept is significantly different from zero, the null hypothesis of no difference between the two object types can be rejected. However, in some experimental conditions, displays contained not only one but two unrelated objects (see Fig 1). In the corresponding GLMMs, the intercept under the null hypothesis (H_0_: *b = b*_0_) was adjusted accordingly: *b*_0_ = logit(1/3) = -0.693. In this case, the significance test was performed using the difference score *d* = *b*–*b*_0_ rather than *b*.

Mixed models are statistical models that incorporate both fixed-effects parameters and random effects. Random effects allow for capturing variance attributed to the randomness of participant and item sampling. Due to the counterbalancing, in the relevant trials subjects and item displays are partially crossed random effects. For example, in the data analysed from Experiment 1 there were 120 unique item displays (consisting of four objects each), and each display was seen by 10 subjects.

To select an appropriate random-effects structure for the LMMs, we pursued a data-driven approach [44]. For each of the 15 analyses (3 continuous dependent variables × 5 experimental conditions), four models that differed in their random effects structure were compared. The first model included random intercepts for subjects only. Given the contrast coding used, including this intercept captures the degree to which subjects vary in their response to semantically related objects. The second model added random intercepts for item displays. The third model added by-subject random slopes for “object type.” The fourth model included random intercepts and slopes for subjects and item displays, i.e., the maximal random effects structure [45]. In case of more complex structures, the estimation algorithm did not always converge to a solution, probably because the model was too complex relative to the amount of data available. The four models and/or the ones that converged were compared using likelihood ratio tests. The log-likelihood increases with goodness of fit. The Akaike Information Criterion (AIC, decreases with goodness of fit) corrects the log-likelihood statistic for the number of estimated parameters. The Bayesian Information Criterion (BIC, decreases with goodness of fit) additionally corrects for the number of observations [46]. Taken together, we used forward model selection to test whether adding a random effect significantly improved the model fit (with improvement indicated by a smaller BIC).

The binomial logit mixed models testing the probability of immediate fixation were varying-intercept models with no predictors (cf. [47]). In the first model, the intercept was allowed to vary by subject. In the second model, random intercepts for item displays were additionally included. We report the model with the smaller BIC. See OSF documentation for details on the full set of (G)LMMs.

## Results

### Extrafoveal processing

We report three different measures to make inferences about the level of information processing available in extrafoveal vision. Our first response variable is *the latency to first fixation* on the object, which is defined as the time elapsed between the onset of the display and the first fixation on the object. The order in which objects are fixated is irrelevant for calculating this temporal measure. The latency to first fixation indicates the potency of an object in attracting early attention using extrafoveal vision. Objects that are fixated earlier are assumed to be more potent in attracting attention than are other objects in the display. Critically, if semantic processing takes place in extrafoveal vision, then semantically related objects should be fixated earlier than unrelated objects. Second, *the probability of immediate fixation* allows us to examine whether the semantic relationship between the spoken target word and the semantically related object can be detected immediately upon first fixation in the display. Third, we report *the mean amplitude of the first saccade into the object*. This tells us whether saccade target selection was based on information in parafoveal (< 5°) and peripheral (> 5°) vision as opposed to near-foveal vision (cf. [15]).

#### Latency to first fixation

**Fig 2:**
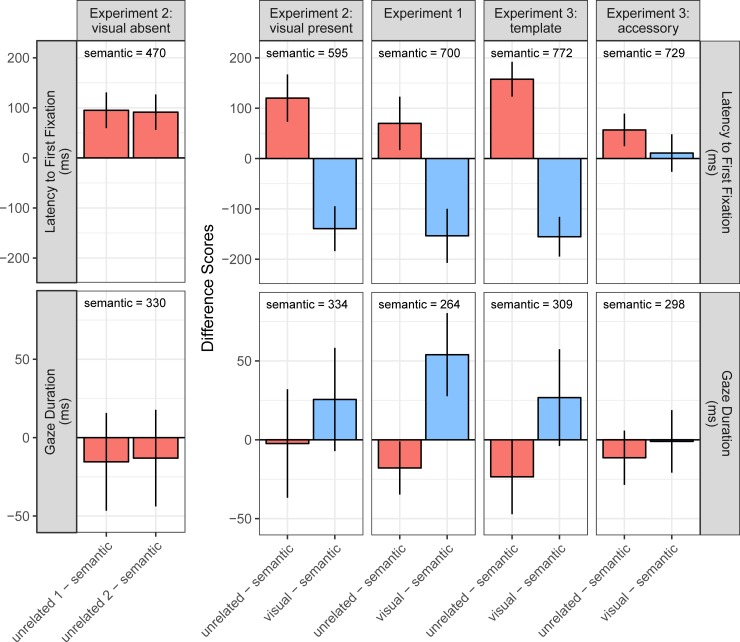
Results for extrafoveal and foveal semantic processing across three experiments. The two main continuous response variables are organized by row. In a given row, each column depicts data from a different experiment or experimental condition. Each facet summarizes the fixed-effects results from the relevant LMM. In the statistical models, the intercept represents the estimate for the semantically related object, and this numeric value is included in the figure panels. The bar charts show the difference scores. The zero line represents the semantically related object as the reference category. The red bars, comparing unrelated to semantically related objects (unrelated—semantic), show the disadvantage of unrelated over semantically related objects, which is equivalent to an advantage of semantically related over unrelated objects. The blue bars, comparing visually related objects to semantically related objects (visual—semantic), show the additional advantage of visually related objects over semantically related objects. Error bars are 95% confidence intervals (CI = ± 1.96 × SE); thus, effects are significant when the error bar does not include 0.

For the latency to first fixation, we consistently observe across the different data sets an advantage of semantically related objects over unrelated objects. In the LMMs, this was shown as a significant positive difference score (unrelated-sem, see Table 1 for values, and Fig 2: red bars, first row). Thus, the eyes went faster to a semantically related than to an unrelated object. In addition to the unrelated and semantically related objects, the search displays also included a visually related object (except for the ‘visual absent’ condition in Experiment 2). Accordingly, the second contrast describes the difference between visually and semantically related objects (vis-sem, see Table 1 for values, and Fig 2: blue bars, first row). The default pattern is that the latency to first fixation was significantly shorter for visually than for semantically related objects. Only in the accessory condition of Experiment 3, where the spoken word was no longer relevant for the search, this difference disappeared.

**Table 1:** Linear mixed models fitting measures of extrafoveal and foveal processing for visual search data from three experiments: Means (b), standard errors (SE), and test statistics (t-values) for fixed effects.

		Experiment 1			Experiment 2: vis absent			Experiment 2: vis present			Experiment 3: template			Experiment 3: accessory		
		sem	u- sem	vis-sem	sem	u1-sem	u2-sem	sem	u- sem	vis-sem	sem	u- sem	vis-sem	sem	u- sem	vis-sem
**Latency to** **first fixation**	*b*	700	70	-154	470	95	91	595	120	-139	772	158	-155	729	57	11
	*SE*	26	27	27	17	18	18	23	24	23	22	18	20	22	17	19
	*t*	27.04	2.59	-5.60	27.82	5.22	5.07	25.58	5.02	-6.13	35.22	8.93	-7.73	33.55	3.45	**0.58**
**Saccade amplitude**	*b*	10.04	0.07	-0.75	6.54	0.49	0.49	7.11	0.41	-0.81	10.29	0.34	-0.66	9.76	0.23	0.04
	*SE*	0.23	0.15	0.17	0.18	0.24	0.24	0.16	0.16	0.16	0.19	0.14	0.15	0.21	0.14	0.16
	*t*	44.04	**0.43**	-4.49	35.83	2.04	2.08	43.47	2.49	-5.19	54.83	2.53	-4.31	47.19	**1.65**	**0.28**
**Gaze duration**	*b*	264	-18	54	330	-15	-13	334	-2	26	309	-23	27	298	-11	-1
	*SE*	10	9	13	19	16	17	26	18	17	16	12	16	13	9	10
	*t*	26	-2.08	4.02	17.28	**-0.97**	**-0.83**	12.95	**-0.13**	**1.53**	19.43	-1.94	1.71	23.67	**-1.3**	**-0.1**

*Note*. Non-significant coefficients are set in bold (|*t*| < 1.96). Sem stands for semantically related objects and vis for visually related objects. The average was taken of the unrelated objects (denoted as u), except for the “visual absent” condition in Experiment 2 where the data is being displayed for each unrelated object separately (u1 and u2).

#### Probability of immediate fixation

**Fig 3:**
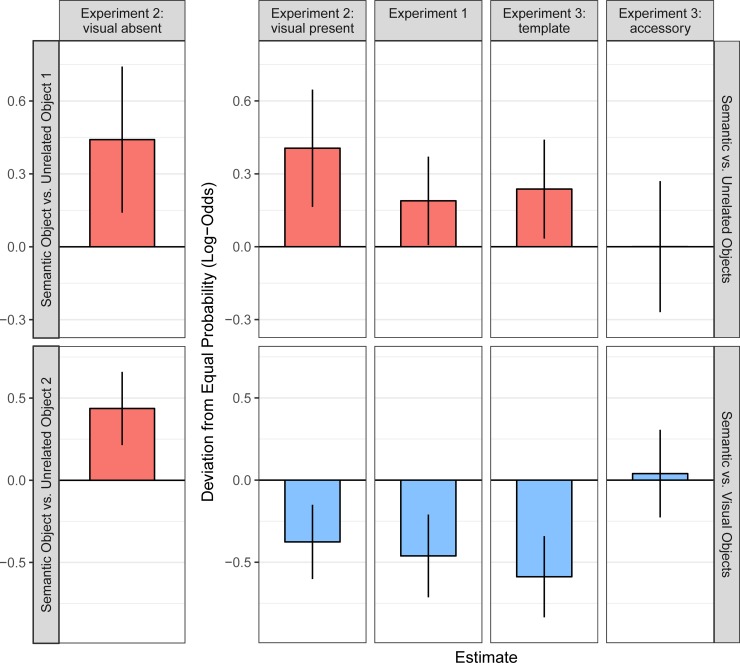
Results for the probability of immediate fixation across three experiments. Each column depicts data from a different experiment or experimental condition. For each of these, two separate intercept-only GLMMs were fitted, the first one comparing semantically related objects with unrelated objects (top row, red bars), and the second one comparing semantically related objects with visually related objects (bottom row, blue bars). The two subplots on the left show the results for a condition from Experiment 2 in which the visually related object was replaced with a second unrelated object. In each facet, the height of the bar represents the estimate for the fixed-effect intercept. The zero line represents the intercept under the null hypothesis. In the analyses represented by the red bars, a positive estimate corresponds to a higher probability for semantically related than for unrelated objects. In the analyses represented by the blue bars, a negative estimate corresponds to a lower probability for semantically related than for visually related objects. Error bars are 95% confidence intervals; thus, the effect is significant if the error bar does not include 0.

The analyses of the latency to first fixation indicate that semantically related objects attract attention in extrafoveal vision, compared to unrelated objects. In the light of these findings, the question arises whether this can happen *immediately*, i.e. upon first fixation. To this end, we analysed the probability of immediate object fixation. For each experiment or experimental condition, separate intercept-only binomial logit mixed models were specified to compare semantically related objects to (1) unrelated objects, and (2) visually related objects (Table 2, Fig 3). As detailed in the Methods section, the model intercept estimates the proportion of cases in which the first saccade was directed to semantically related objects. In the accessory condition of Experiment 3, where the word was irrelevant for the search, there was no significant difference between semantically related objects and unrelated objects; there was also no significant difference between semantically related and visually related objects. In all other conditions, for all experiments, semantically related objects were more likely to be selected with the very first saccade than unrelated objects. In the GLMMs, this was shown as a significant positive estimate for *b* and/or *d* (Table 2, Fig 3: red bars). Moreover, semantically related objects—when present—were selected less often than visually related objects (Table 2, Fig 3: blue bars). Taken together, the very first fixation was determined by both visual as well as semantic features of the objects in relation to the spoken target word.

**Table 2:** Intercept-only GLMMs fitting the probability of immediate fixation for visual search data from three experiments: Null hypothesis (b_0_), means (b), standard errors (SE), and test statistics (z-values and p-values) for the fixed effect intercept.

Experiment	Test	*b*_0_	*b*	d	*SE*	*z*	*p*
Experiment 1	semantic vs. unrelated	logit(1/3) = -0.693	-0.5	0.19	0.09	2.03	0.042
Experiment 1	semantic vs. visual	logit(1/2) = 0	-0.46		0.13	-3.6	< .001
Experiment 2: visual absent	semantic vs. unrelated 1	logit(1/2) = 0	0.44		0.15	2.87	0.004
Experiment 2: visual absent	semantic vs. unrelated 2	logit(1/2) = 0	0.44		0.11	3.84	< .001
Experiment 2: visual present	semantic vs. unrelated	logit(1/2) = 0	0.41		0.12	3.29	0.001
Experiment 2: visual present	semantic vs. visual	logit(1/2) = 0	-0.38		0.12	-3.26	0.001
Experiment 3: template	semantic vs. unrelated	logit(1/3) = -0.693	-0.46	0.24	0.1	2.28	0.022
Experiment 3: template	semantic vs. visual	logit(1/2) = 0	-0.59		0.13	-4.66	< .001
Experiment 3: accessory	semantic vs. unrelated	logit(1/3) = -0.693	-0.69	0	0.14	0	0.998
Experiment 3: accessory	semantic vs. visual	logit(1/2) = 0	0.04		0.14	0.29	0.773

*Note*. H_0_: *b = b*_0_; d = *b*–*b*_0_; *z* = *b* / *SE* if d = *b*, *z* = d / *SE* if d ≠ *b*

#### Incoming saccade amplitude

The incoming saccade amplitude denotes the amplitude of the saccade that first entered the object. In the LMM the fixed-effect intercept represents the mean amplitude (°) of saccades entering semantically related objects. In Experiments 1 and 3, this amplitude was about 10° (Table 1). In Experiment 2, the mean amplitudes of the saccades entering semantically related objects were smaller at approximately 7°, due to the different display dimensions. Taken together, the saccade amplitude data suggest that observers could determine the semantic relationship between the spoken target word and the relevant object well outside the fovea, in the range typically labelled as the periphery (> 5°).

The LMMs further tested whether the amplitude of the incoming saccade was affected by Object Type. This was not the case for the accessory condition in Experiment 3. But for all other data sets where visually related pictures were included in the display, the incoming saccade was significantly shorter for the visually related object than for the semantically related object. Furthermore, saccades into semantically related objects were in turn significantly shorter than for unrelated objects in the both the ‘visual present’ and ‘visual absent’ conditions of Experiment 2, and in the template condition in Experiment 3, but not in Experiment 1. Overall, the incoming saccades were the smallest for visually related pictures and the largest for the unrelated pictures (with the semantically related pictures falling in between). Note that this is to be expected: unrelated pictures were fixated later in time; thus, these fixations were more likely coming from another object rather than from central fixation. The distance between objects was larger than the distance between objects and central fixation.

### Foveal processing

For completeness, we also analysed whether the visual and semantic relationships affected object processing once the object was fixated. Specifically, we explored whether fixation times were affected by the relationship between the spoken target word and objects in the display. To this end, we calculated first-pass gaze duration as a common measure to index the degree of attention allocated to the objects [22]. First-pass gaze duration is defined as the sum of all fixation durations from first entry to first exit [48]. For the data from Experiment 1, there was a systematic ordering such that gaze durations were longest for visually related objects and shortest for unrelated objects, with gaze duration for semantically related objects falling in between; these effects were marginally significant for the template condition in Experiment 3 (Table 1; Fig 2, second row). In Experiment 2, gaze duration was not reliably modulated by Object Type; the same was true for the accessory condition in Experiment 3.

## Discussion

In the past, researchers have been debating whether the semantic properties of objects are available for attentional guidance from extrafoveal vision. We find that, across different datasets, semantically related objects were fixated earlier in time than unrelated objects. Moreover, observers directed their very first saccade more often to semantically related objects than to unrelated objects. Importantly, with 7–10° on average, the amplitudes of the saccades that first entered the semantically related object were large, confirming that these objects were processed in peripheral vision (typically > 5°). Collectively, these findings demonstrate that participants were able to quickly activate and process semantic information about objects in the extrafoveal visual field and use it to guide the first eye movement. Finally, the semantic effects were observed with and without visual competition and disappeared when the semantic representations were no longer relevant to the search task.

Our results corroborate and extend earlier findings by Moores et al. [25] and Belke et al. [26]. They too found that, on target-absent trials, participants were significantly more likely to look first to an associate to the target than to any of the other objects in the display. The authors analysed first saccade landing points [25] or first fixation locations [26] as a measure of the initial deployment of attention, but the issue of extrafoveal attentional guidance by object semantics was not central to their investigation. Moreover, their design involved repeated exposure to the same semantic associate. Here, we replicate and extend their findings while at the same time controlling for visual and linguistic factors and avoiding any stimulus repetitions. Importantly, the current results cannot be explained by low-level bottom-up factors. First, all stimuli were highly controlled: we matched the semantically related objects and the unrelated objects overall on low-level visual features such as luminance, visual complexity, and size [29]. Second, semantic and visual relatedness were dissociated. The current results are therefore unlikely to be explained by a visual relationship. Third, effects disappeared in the condition in which the word was irrelevant for the search task (i.e., the accessory condition in Experiment 3, which used the exact same stimuli as the conditions in which we did find effects). This also suggests that the observed effects were not driven by low-level visual features (or any other non-task-related factors), but by the relative importance of the semantic information for the observer.

The present results are relevant to a long-standing debate in the field of scene perception and search, regarding biases towards objects that are semantically incongruent with the overall meaning of the scenes in which they occur (see refs. [7] and [8] for reviews). Studies using task instructions ranging from free viewing [19] over scene memorization [10, 18] to change detection [14] have generated mixed evidence. In particular, visual search studies using everyday scenes have found either more efficient guidance towards inconsistent objects [13], towards consistent objects [18], or no difference between consistent and inconsistent objects [20]. Moreover, Loftus and Mackworth’s classic “octopus in a farmyard” study [15], in which observers’ gaze was often immediately directed to semantically informative peripheral regions, has been subjected to strong criticism, as the early effects observed in this study may have been due to visual salience rather than object-scene semantics (see ref. [22] for a review).

Our experiments circumvent some of the conundrums associated with whole scene displays. We note, however, that differences in the way these experiments are designed may make any such comparison difficult. Notably, in the present type of experiments, objects are assessed in relation to a spoken target word, while in experiments using images of real-world scenes, the critical objects are assessed in relation to the scene context which has to be perceived in itself. Our results thus provide an existence proof that object semantics can be activated rapidly enough to influence the planning and execution of the first saccadic eye movement to objects placed in peripheral vision. The theoretical implication of such immediate semantic “pop-out” is the conclusion that objects can be processed to a semantic level over a large area of the visual field in a short amount of time (i.e., within a single fixation). In the context of scene viewing, the analogous (but still controversial) finding [15] would be that the overall meaning, or “gist” of a scene can be rapidly apprehended and that the scene gist is analysed to a very high level using peripheral vision. Finally, the results also connect to similar debated research on reading. In the literature on eye guidance in reading, the nature of attention allocation (serial or parallel) is currently an issue of much contention [49, 50]. Interestingly, any evidence that semantic information is acquired from the parafovea is generally taken to be at odds with models that posit serial processing of words. By comparison, these issues are not well explored in object and scene perception, but they bear less potential for contention [51].

Most of our experiments used displays that contained not only a semantically related object, but also an object that was visually related to the target word. Overall, attentional capture was stronger for visually related objects than for semantically related objects. This suggests that, overall, visual representations are relatively more important for attentional orienting than semantic representations, at least under the current circumstances. Moreover, it relates to earlier visual search studies showing that verbal instructions are less efficient in guiding attention than pictorial instructions [52–57]. This makes sense given that the task is, by definition, visual in nature, so it is beneficial to activate a strong visual representation when available. Indeed, when the spoken word was irrelevant for search, most effects were no longer observable, indicating that the activation of these representations, both visual and semantic, is flexible [28]. Future research could explore whether there are conditions under which semantic representations dominate over visual representations.

Finally, we believe the results are important for models of visual attention (cf. [58]). So far, visual attention models have focused on low-level visual features, and have not incorporated the meaning of objects as a property on which extrafoveal attentional guidance can be based [59–63], or have even explicitly excluded object meaning from the list of properties available for attentional guidance [64]. This is not to say that such models deny the influence of meaning altogether. They have increasingly incorporated the semantic context of the entire scene, a context which steers attention towards locations that are likely to hold the target object (e.g. biasing attention towards pavement areas when looking for people in an urban scene, or a kitchen work top when looking for a cutting board) [62, 65, 66]. However, where these effects reflect spatial biases on the basis of overall scene gist, we demonstrate attentional guidance on the basis of individual object meaning, without any further context other than the sought-for target object.

Last but not least, we chose (generalized) linear mixed models to analyse our data. The many advantages of (G)LMMs are well documented [31–33, 36]. For example, they are better than ANOVAs in handling unbalanced data [33]. For one, this refers to unbalance due to the experimental design: our displays typically contained two unrelated objects, but only one semantically and one visually related object. More importantly, participants in eye-tracking experiments oftentimes generate unbalanced data due to their gaze behaviour. Our observers did not always fixate all objects in a display; interestingly, unrelated objects were “skipped” more often than semantic and visual objects. In an ANOVA, this imbalance (and subsequent difference in reliability) is ignored by averaging the dependent variable to a single value per experimental condition [33].

In conclusion, results from three experiments using arrays of standalone objects demonstrate that semantically related objects can attract attention outside foveal vision, and that this can happen very rapidly. The results imply that observers could determine the semantic relationship between the target word and the related object in the display based solely on extrafoveal information obtained within a single fixation, and that semantic information could exert an immediate effect on eye-movement control.
