# Correction: Extrafoveal attentional capture by object semantics

**DOI:** 10.1371/journal.pone.0218502

**Published:** 2019-06-12

**Authors:** The PLOS ONE Staff

Due to a typesetting error, the Data Availability statement for this paper is incomplete. The publisher apologizes for the error.

The complete Data Availability statement is: All data and analysis files are available from the Open Science Framework: http://dx.doi.org/10.17605/OSF.IO/36RQC (https://osf.io/36rqc/).
